# Treatment of bloodstream infections in ICUs

**DOI:** 10.1186/1471-2334-14-489

**Published:** 2014-11-28

**Authors:** Jean-François Timsit, Jean-François Soubirou, Guillaume Voiriot, Sarah Chemam, Mathilde Neuville, Bruno Mourvillier, Romain Sonneville, Eric Mariotte, Lila Bouadma, Michel Wolff

**Affiliations:** UMR 1137, INSERM, IAME, F-75018 Paris, France; UMR 1137, Univ Paris Diderot, IAME, Sorbonne Paris Cité, F-75018 Paris, France; Medical and infectious diseases ICU, AP-HP, Hôpital Bichat, F-75018 Paris, France

**Keywords:** Bloodstream infection, Healthcare associated infections, Bacterial resistance, Intensive care unit

## Abstract

Bloodstream infections (BSIs) are frequent in ICU and is a prognostic factor of severe sepsis. Community acquired BSIs usually due to susceptible bacteria should be clearly differentiated from healthcare associated BSIs frequently due to resistant hospital strains. Early adequate treatment is key and should use guidelines and direct examination of samples performed from the infectious source. Previous antibiotic therapy knowledge, history of multi-drug resistant organism (MDRO) carriage are other major determinants of first choice antimicrobials in heathcare-associated and nosocomial BSIs. Initial antimicrobial dose should be adapted to pharmacokinetic knowledge. In general, a high dose is recommended at the beginning of treatment.

If MDRO is suspected combination antibiotic therapy is mandatory because it increase the spectrum of treatment. Most of time, combination should be pursued no more than 2 to 5 days.

Given the negative impact of useless antimicrobials, maximal effort should be done to decrease the antibiotic selection pressure. De-escalation from a broad spectrum to a narrow spectrum antimicrobial decreases the antibiotic selection pressure without negative impact on mortality. Duration of therapy should be shortened as often as possible especially when organism is susceptible, when the infection source has been totally controlled.

**Electronic supplementary material:**

The online version of this article (doi:10.1186/1471-2334-14-489) contains supplementary material, which is available to authorized users.

## Introduction

Bloodstream infections (BSI) are a major cause of morbidity and mortality worldwide [1]. These infections may arise secondarily to localized infection at a specific body site, or may be classified as primary when no focus is identified. Severe sepsis, septic shock, and multisystem organ dysfunction related to BSI frequently require admission in ICU for appropriate management [2]. BSI, as a complication of critical illness, occurs in approximately 5% of all patients admitted to ICUs [3, 4]. The case-fatality rate associated with BSI reaches 35-50% when associated with admission to ICU [1].

The mainstay of therapy for patients with bacteremia remains antimicrobial therapy, together with the optimal management of its consequences (e.g., shock or metastatic suppurative complications), and surgical treatment of an infection site (e.g., debridement, abscess drainage, or removal of intravascular devices), when appropriate.

Considering the paucity of new antimicrobials in the industry pipeline, and the emergence of strains resistant to recent antimicrobials , enhanced efforts must be made to decrease selection pressure to antimicrobials [5]. To contain the worldwide problem of antimicrobial resistance, intensivists have two major tools with regards to antimicrobials treatments: [1] antimicrobial de-escalation, and [2] shortened duration of antimicrobial therapy. Available data suggest that the treatment should be shortened systematically [6] or based on the evolution of procalcitonin levels [7, 8].

We will review the main elements that should be taken into account for treating BSIs in ICU (figure).

### At the initiation of antimicrobial therapy

**Figure 1:**
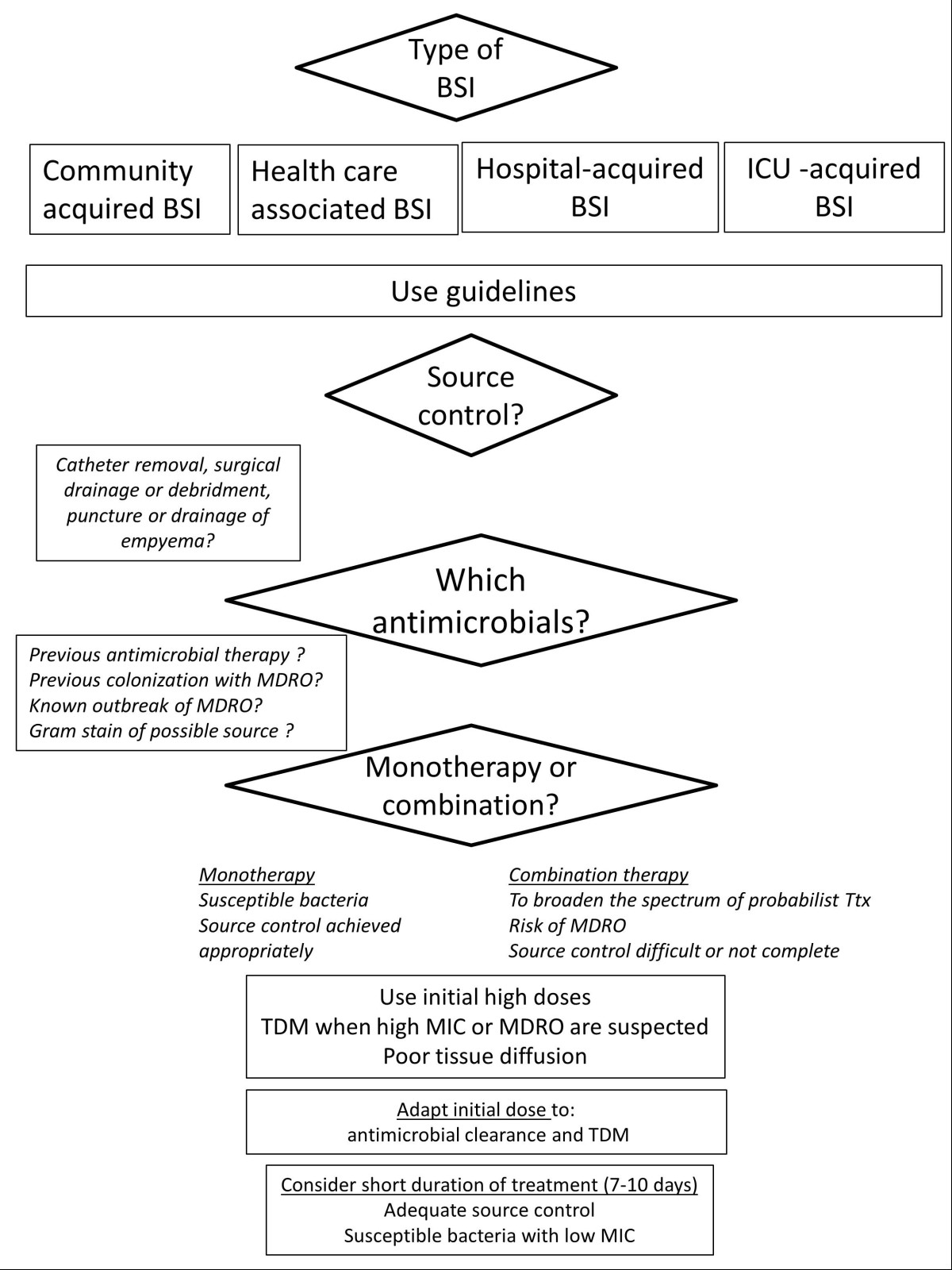
**Decision tree including main determinants guiding the choice of the most appropriate antimicrobial therapy.** (BSI: bloodstream infection; MDRO: multiresistant drug organism; TDM: therapeutic drug monitoring; MIC: minimum inhibitory concentration).

Early appropriate antibiotic therapy is a fundamentally important aspect of therapy of patients with BSI. Adequate treatment requires [1] that all organisms isolated from blood are susceptible *in vitro* to the antimicrobials chosen; [2] a proper route and dose, and [3] an early administration after blood cultures collection. In a recent large hospital cohort study, Retamar *et al* showed that, even fully adjusted on confounding factors, inadequate antibiotic therapy within 24 hours was associated with a significant OR of 3 for the 14-day mortality and 1.70 for the 30-day mortality, respectively [9]. In a large cohort of septic patients, Kumar *et al* found that inadequate therapy within 6 hours after onset of hypotension was associated with an increase of the risk of death by more than 5-fold in case of septic shock, and by more than 9-fold if a BSI is associated to the septic shock [10]. In a very recent analysis of the surviving sepsis campaign dataset involving 17,990 patients with severe sepsis who received appropriate antimicrobials, after a careful adjustment on severity, geographic region, admission source, the mortality increased steadily by about 1 percent per hour from 24.6% when treatment was adequate within one hour to 33.1% when treatment was adequate after the 6^th^ hour [11]. In the Eurobact study [12], an increased mortality was observed among patients who had hospital-acquired bacteremia and were hospitalized in ICU but never appropriately treated (adjusted odds ratio 1.56; 95% CI 1.04-2.35, p = 0.03). Conversely, a very early treatment (<one day after having drawn the first positive blood culture) was not associated with a decreased risk of death as compared to rather delayed appropriate treatments (<2 days and < 5 days). Some kind of underdosage, associated with the increase of the volume of distribution and the paradoxical increase in glomerular filtration frequently observed in case of septic shock, may explain this result.The initial antimicrobial therapy is necessarily empiric, targeting the most likely etiologic pathogens. When risk factors of antibiotic resistance are identified in patients with a serious infection, broad spectrum antimicrobials should be prescribed. The determinants of first-choice molecules when empirical treatment is described in Figure 1.

In a *post hoc* analysis among emergency department patients undergoing a quantitative resuscitation protocol for septic shock, Puskarich *et al* found that [13] the risk of death increased when antimicrobials were delayed until after the onset of shock (odds ratio = 2.59 95% CI 1.17-5.78). This suggests that, if an aggressive resuscitation is performed early, the timing of initial antibiotic therapy may have lesser importance.

On the opposite, when antimicrobial are started in the first hours of severe sepsis, the recent Protocolized Care for Early Septic Shock (ProCESS) trial have found no difference in mortality between an aggressive therapy of organ hypoperfusion that require the placement of a central venous catheter, administration of inotropes, or blood transfusions, a protocol-based standard therapy that did not, or an up-to-date usual care [14].

While controversy exists regarding which elements of sepsis resuscitation bundles are most beneficial, it is intuitive that good principles of care dictate that appropriate antimicrobials be prescribed promptly in the setting of severe infection.

### Epidemiology of resistant bacteria

Epidemiological data about spread of MDRO has been updated by the WHO organization very recently. These data clearly found a global increase in the risk of bacterial resistance mainly gram negative bacteria with great variability between countries. The global report updated in april 2014, is available in the WHO website (http://www.who.int/drugresistance/documents/surveillancereport/en/; accessed august 22^nd^ 2014). These general data collected mainly by hospital lab imperfectly reflect the pathogen responsible for bacteremia in ICU patients.

For community-acquired infections, recovered microorganisms are mainly *Streptococcus pneumoniae* and *Staphylococcus aureus* for Gram positive, and *Escherichia coli* for Gram negative. The betalactams active against these bacterias did not change in the past decade. However, extended spectrum beta-lactamase (ESBL)-producing *E. coli* and community acquired MRSA are more and more reported.

The fecal carriage of ESBL *E. coli* (mainly CTX M-15), which was unknown before the turn of the millennium, has since increased significantly everywhere, with developing countries being the most affected (Southeast Asia, Eastern Mediterranean region, Western Pacific region). Intercontinental travels may have emphasized and globalized the issue [15]. Among European subjects with traveler’s diarrhea, returning from diverse overseas areas, ESBL-*E. coli* carriage rates were observed in 1 out of five. These rates increased significantly after travel to Egypt, India, Southeast Asia, Thailand, and the Middle East [16]. In a prospective case-control study performed in 2008, European travelers had a 23% ESBL-*E. coli* carriage rate, which was significantly more than the 4% found in non travelers. Upon return from India, Africa, or Asia, the ESBL-E carriage rate reached 46% [17]. The duration of carriage after travel seems to be relatively short, lasting only a few weeks. The practical consequences of these epidemiological changes is that empirical therapy of septic shock probably due to Gram negative organisms (i.e., urinary tract infections or intra-abdominal infections) after returning from endemic areas must cover ESBL *Enterobacteriaceae*.

Community-acquired methicillin-resistant S. aureus (CA-MRSA) is another matter of concern. CA-MRSA responsible of community acquired pneumonia (CAP) is very uncommon in Europe [18] (less than 1% of CAP) but much more frequent (8-10%) in USA. MRSA treatment in case of CAP is not recommended [19], but should be borne in mind in case of severe necrotizing CAP with hemoptysis and leucopenia, often preceded by influenza infection [20].

For nosocomial and health-care associated (HCA) bloodstream infections, resistance profile is highly dependent on national and local epidemiology. Hospital-acquired and HCA BSIs are associated with increased incidence of multi-resistant drug microorganisms (MDRO), such as MRSA, ESBL producing *Enterobacteriaceae*, *Acinetobacter baumannii*, and *Pseudomonas aeruginosa*. In these cases, it is more difficult for empiric treatment to be appropriate, especially in patients admitted in the ICU with a major incidence of multidrug-resistant microorganisms [21, 22]. In these cases, in addition to applying guidelines recommendations, it is indispensable to know the local flora predominant in each area before initiating empiric antibiotic treatment.

Table 1 displays the distribution of organisms from recent studies investigating the epidemiology of BSIs in ICUs. The most important change in the epidemiology of ICU-related BSI in recent years is the emergence of highly resistant organisms, particularly extensively resistant Gram negative bacteria including *P. aeruginosa*, *A. baumannii* and *Klebsiella pneumoniae*[26–28].

**Table 1:** **Microorganisms recovered from BSIs in ICU in the main recent studies**

Author	Marra 2011[23]	Prowle 2011[4]	Valles 2011[24]			Corona 2010[25]			Tabah 2012[12]		
(countries)	(Brazil)	(Australia)	(Spain, Argentina)			(Europe, Australasia, South America, Asia)			(Europe, South America, China, Canada)		
Type of BSI (number of cases)	HAB (1196)	ICU-AB (330)	CAB (343)	HCAB (131)	HAB (252)	CAB (431)	HAB (351)	ICU-AB (915)	HAB (279)	ICU-AB (877)	% MDRO^b^
*S. pneumoniae*			21.4%			10.9%	1.4%	0.4%	--	--	
*S. aureus*	12.8%	26.7%	10.3%^a^	13.8%^a^	9.6%^a^	22.3%	26.2%	23.6%	16.1%	8.4%	60%
CoNS	16.6%	24.3%	3.5%	6.2%	10.8%	16.5%	19.7%	29.6%	10%	12.7%	--
*Enterococcus* spp.	5.5%	17%	1.5%	4.6%	8.4%	7.2%	9.1%	11.4%	10%	12.9%	43.5%
*E. coli*		Gram negative 28.2%	20.5%	26.9	18.5%	19%	9.7%	5.6%	19%	5.1%	66.7%
*Enterobacter* spp.	5.8%		2.3%	6.9%	3.6%	2.6%	4.8%	6.3%	7.2%	7.6%	50%
*Klebsiella* spp.	11.8%		4.1%	4.6%	6.0%	4.6%	8.6%	8.5%	8.2%	15.2%	90.9%
*Serratia* spp.	3.2%								1.8%	2.6%	69%
*Proteus* spp.	1.8%					3.3%	1.4%	1.5%	1.4%	1.7%	20%
*P. aeruginosa*	10%		1.8%	4.6%	6.4%	6%	10.3%	9.7%	10.4%	12.5%	60%
*Acinetobacter* spp*.*	11.8%					0.9%	5.4%	5.7%	7.5%	15.7%	95.2%
Anaerobes			3.2%	1.5%	1.6%				3.9%	0.7%	0
*Candida* spp.	5.8%	15.5%	1.5%	3.8%	8.4%	4.9%	10.5%	6.5%	7.5%	8.2%	--

Legend: HAB: hospital-acquired BSI, ICU-AB: ICU acquired BSI, HCAB: healthcare-associated BSI, MDRO : multi drug resistant organisms.

a: MRSA represents 3% of CAB, 27.5% of HCAB and 54.1% of HAB.

b: proportion of multidrug resistant organisms for both HAP and ICU-AB.

### Timing of BSI onset versus hospital admission

BSIs are generally classified as either being community-acquired (CA) or nosocomial infections [29]. However, moe and more patients with complex medical problems are now managed in the community setting. Those patients managed in the outpatient setting have infections that share many clinical, microbiologic, and outcome characteristics with nosocomial infections. As a result, a new category of healthcare-associated (HCA) community-onset infections has become widely recognized [30].

Traditional risk factors of HCA infections are [1] residency in a nursing home or long-term care facility in the 30 days before the BSI. [2] hospitalization in an acute care hospital for 48 h or longer in the 90 days before the BSI. [3]. Attended a hospital or dialysis clinic or received intravenous chemotherapy in the 30 days before the BSI. [4] Received intravenous therapy, wound care, enteric nutrition, or specialized nursing care at home, in the 30 days before the BSI but the respective impact of each risk factor has never been fully investigated and probably vary between countries [31].

In hospital settings, many recent publications showed that *S.aureus*, *P aeruginosa* is much more common in HCA-BSI than in CA BSI. *E coli* is more frequent in CA BSI but the rate of ESBL E coli is similar between CA and HCA BSIs [31–37]. To the best of our knowledge there is no study that has compared microorganisms responsible from HCA-BSI and CA or HA BSI admitted in ICUs.

### Patient’s prior antimicrobial history

If a prior antibiotic use is known, it should be considered in conjunction with previous bacterial culture and antimicrobial susceptibility testings, for setting up an appropriate treatment [38]. Most of the time, the prior use of one antibiotic is associated with an increase in the risk of infection with a microorganism resistant to it [39, 40]. In area where the rate of carbapenem-resistant micro-organism is important, previous antimicrobial therapy is of key importance. The duration of exposure to carbapenems (OR 1.079 per day of exposure, 95% CI 1.022-1.139, p = 0.006) and to colistin (OR 1.113 per day of exposure, 95% CI 1.046-1.184, p = 0.001) were independent risk factors for acquisition of CR-GNB bacteremia in a Greek ICU [41]. Indeed, previous antimicrobial therapy strongly modified the gut microbiota, the main reservoir of potential pathogens in ICU patients. As an example, in our ICU, Armand-Lefevre and colleagues [42] found that imipenem resistant Gram negative bacteria (IRGNB) in the digestive flora increased regularly from 5.6% after 1 week to 58.6% after 6 weeks in the ICU. Imipenem exposure was the main risk factor of IRGNB. As compared to patients that did not received imipenem, the odds ratio for colonization was already as high as 5.9 (95% confidence interval [43], 1.5 to 25.7) after only 1 to 3 days of exposure and increased up to 7.8 (95% CI, 2.4 to 29.8) thereafter.

### Source of infection

The source of infection is another key element to choose empirical therapy with two objectives. We need to adapt the choice of antimicrobials to the probable agents responsible for infection and to the diffusion of antimicrobials to infected tissues.

For CA infections, pneumonia is associated with bacteremia in 15% cases. *S. pneumoniae* represents the main cause of bacteremia of respiratory origin, followed by *S. aureus*, as showed by a recent large multicenter ICU cohort study [44]. *Enterobacteriaceae* and *P. aeruginosa* are encountered in less than 5% of the CA pneumonia, mainly HCA-pneumonia, but is associated with bacteremia in about half of the cases [45]. For digestive and urinary tract infection, *E. coli* is by far the main cause of CA BSI.

In the French national network REA RAISIN in 2013 (213 ICUs, 34 278 patients), the main causes of ICU-acquired bacteremia were intravascular catheters (29.2%), lower respiratory tract infections (18.0%) and digestive tract (13.6%). The source of ICU-acquired bacteremia remained unknown in 24.9% cases. In the same network, the main causes of catheter-related BSIs were coagulase negative staphylococci (28%), *S. aureus* (23%), *P. aeruginosa* (12%), *E. coli* (8%) and multiple causes (11.5%) [46].

In a *post hoc* analysis of the Eurobact study among 230 patients with HA-BSI respiratory infections (HABSI requiring ICU admission, n = 40; ICU-acquired, noninvasively ventilated respiratory BSI, n = 30; and ICU-acquired, invasively ventilated BSI, n = 160) were compared to 749 patients with HA-BSI not related to respiratory infections. HA-BSI respiratory infections were more frequently due to Gram negative pathogens (76.3% vs. 56.7%, P < 0.0001), mainly *A. baumannii* (18.3% vs. 10.4%, P = 0.0007), *Klebsiella* spp. (18.7% vs. 11%, P = 0.0013), and were less frequently due to Gram-positive cocci (23.3% vs. 41.2%, P < 0.0001), with the exception of *S. aureus* (11.3% vs. 9.5%, P = 0.39). HA-BSI respiratory infections were more frequently associated with MDRO (53.9% vs. 42.7%, P = 0.0003) [47].

In a retrospective single-center analysis of 96 patients who developed BSI of abdominal origin admitted in ICU, de Waele *et al* showed that secondary peritonitis, intra-abdominal abscesses and cholangitis were the sources of the BSI in the majority of patients. Gram-negative, especially *E. coli,* were frequently recovered. BSI was polymicrobial in 22% of the cases [48]. Based on epidemiological data and guidelines, Table 2 proposed empirical antibiotic therapy according to situations.

**Table 2:** **Suggested empirical antimicrobial treatment of bloodstream infections according to source of infection and place of acquisition (adapted from international guidelines and local pratice)**[49–55]

Site	Pathogens	Suggested empirical antibiotic
**Urinary tract infection**	Enterobacteriacae including	- Ceftriaxone for CAB or ceftazidime for HAB or ICU AB (if suspicion of *P. aeruginosa*)
	*- Escherichia coli*	± aminoglycosides
	Other enterobacteiraceae	*NB: Consider the risk of ESBL and administer a penem in case of hish risk (CA and recent travel to high risk countries, other situations in case of previous colonization, invasive procedure use, previous antibiotic therapy especially in case of septic shock)*
	*Pseudomonas aeruginosa*	
	*Enterococcus* sp.	
	*Staphylococcus* sp.	
**Intra-abdominal sepsis**	Gram negative bacilli including	- Piperacillin-tazobactam
	*- Escherichia coli* (all BSIs)	- Cephalosporin active against *P. aeruginosa* + metronidazole
	- other enterobacteriaceae (all BSIs)	- Carbapenem (high-risk patients)
	*- Pseudomonas aeruginosa (HA and*	± fluconazole
	*ICU BSI)*	± aminoglycoside
	Gram positive Cocci including	
	*- Enterococcus* sp*.*	
	Anaerobes including *Bacteroides*	
	*Candida*	
**CA and HCA pneumonia**	S pneumoniae (CA BSI)	- Third generation cephalosporin (macrolides in case of suspicion of intracellular bacteria will be added initially).
	S aureus (HCA BSI)	
	E coli (HCA BSI)	- NB: In case of HCA, consider the risk of MRSA or ESBL E coli.
**Hospital acquired pneumonia**	Enterobacteriacae	- Beta-lactam active against *P. aeruginosa* ± aminoglycoside ± glycopeptide or linezolid if suspicion of methicillin-resistant *Staphylococcus aureus*
	*Pseudomonas aeruginosa*	
	*Staphylococcus aureus*	
	*Streptococcus pneumoniae*	
	*Haemophilus influenzae*	
**Ventilator-associated pneumonia (no risk factor for multi-drug resistant pathogen)**	*Staphylococcus aureus*	- Third generation cephalosporins
	*Streptococcus pneumoniae*	± aminoglycosides or fluoroquinolones
	*Haemophilus influenzae*	
	Anaerobes	
**Catheter-related infection**	*Staphylococcus* sp.	- Glycopeptide or linezolid + beta-lactam active against *Pseudomonas aeruginosa*
	Enterobacteriacae	*NB: Consider the risk of candidaemia in case of previous Candida colonization,*
	*Pseudomonas aeruginosa*	
	*Candida sp.*	

### Mono- or bi-antimicrobial therapy?

Although combination therapy has been controversial in regard to a synergistic effect, it is becoming increasingly important to achieve adequate empirical therapy. Aminoglycoside use is associated with an increased risk of impaired renal function; more generally, the use of combination of antimicrobial exposed to more iatrogenic and allergic complications than a single drug. The impact of combination therapy as compared to monotherapy in resistant bacteria selection from the gut flora is also debated. Theoretically, and in experimental studies, combination therapy exposes the gut microbiota to a highest antibiotic selection pressure than monotherapy. However, in a randomized trial among ICU patients with severe sepsis, a combination of meropenem and moxifloxacin was associated to less emergence of resistance at the infection site to meropenem and aminoglycosides than meropenem alone [56]. Underdosage of antimicrobials and poor pharmacodynamic are certainly the main risk factors for bacterial resistance emergence among Gram negative microbiota.

In a retrospective propensity matched study, in case of septic shock, Kumar *et al* found a 23% reduction in the risk of death with the use of immediately adequate combination therapy as compared to immediately adequate monotherapy [57]. But in a cohort of 593 ICU patients with *P aeruginosa*, BSI, there was no demonstrated benefit of a combination therapy if the monotherapy is adequate [58]. In a cohort of 760 patients with severe sepsis or shock, inappropriate initial therapy increased the risk of death (adjusted OR: 2.3); combination therapy decreased the rate of inappropriate therapy from 36% to 22.2% [59].

The appropriate combination is also debated. The benefit of combination with aminoglycosides has not been found in the study by Kumar *et al.*[57]. On another cohort of Gram negative bacteremia, combination with aminoglycosides improved the appropriateness of antimicrobial therapy in episodes due to ESBL or AmpC producing *Enterobacteriaceae* and to *P. aeruginosa*. Combination with aminoglycosides also improve prognosis of patients with neutropenia and septic shock [60].

### Right dosage

Prescription of an antibiotic for which the micro-organism is supposed to be susceptible *in vitro* is not synonymous of efficacy. It is especially the case for the most severe patients (with increase in the volume of distribution of antimicrobials) and for the less susceptible bugs, for which available antimicrobials have MICs at the border of susceptibility breakpoints.

Pharmacokinetic/pharmacodynamic (PK/PD) targets need to be achieved for decreasing the risk of treatment failure and selection of resistant pathogens [61].

The main pharmacodynamic properties that correlate with efficacy for largely used antimicrobials are the time above the MIC for beta-lactams and carbapenems, the ratio maximal concentration/MIC (Cmax/MIC) for fluoroquinolones, aminoglycosides, daptomycin, and colistin, and the area under the curve above the MIC (AUIC) for glycopeptides.

Estrely *et al*[62] studied the impact of MICs to carbapenems of multiresistant Gram negative bacteria (*P. aeruginosa*, *A. baumannii* and Beta-lactamases producer *Enterobacteriaceae*) on the outcome of 71 patients with BSIs. Overall, 52 patients survived, and 19 died. This study revealed that patients with organisms that had a MIC of > 4 mg/L had worse outcomes than patients whose isolates had a MIC of <2 mg/L, even after adjustment for confounding variables (16.1% versus 76.9%; P < 0.01). Indeed, it has been demonstrated that beta-lactams used in critically ill infected patients achieved infrequently their pharmacodynamic target. Usual targets for beta lactams are free antibiotic concentration above the MIC (fT > MIC) of the pathogen at both 50% and 100% of the dosing interval. In the recent DALI study, involving 248 patients with infection, 16% of the beta-lactam administration did not achieve the 50% target and 32% did not achieve the 100% target. Importantly, a positive clinical outcome was associated with 50% and 100% fT > MIC (OR = 1.02 and 1.56, p < 0.03) [63]. In practice, we should know the MIC50 and MIC90 of the suspected pathogens, and ask for specific MICs after pathogen identification, especially for MDRO and extensively resistant pathogens.

For hydrophilic antibiotics such as beta-lactams, aminoglycosides, glycopeptides or colistin, the increase in the volume of distribution observed in severe patients leads to a decrease of the antibiotic concentrations. Furthermore, in young people without immediate renal dysfunction, a paradoxical increase in the glomerular filtration is observed. Both mechanisms argue for the administration of an important initial dose of antimicrobials (loading dose), in order to reach pharmacodynamic targets at the initiation of treatment (suggested initial doses are in Table 3). For lipophylic antibiotics such as fluoroquinolones, the volume of distribution is grossly unchanged and clearance depends on hepatic function.

**Table 3:** **Suggested intravenous of initial selected antibiotic doses in critically ill patients during the first 24 hours of treatment of bacteremia with severe sepsis in ICU (to be adapted in case of kidney injury and renal replacement therapy) (adapted from**[61, 63–66]

Antibiotic class	
Aminoglycosides	Amikacin 25 mg/kg
	Gentamicin 7 mg/kg
	Interval administration and doses adjusted according to TDM
Fluoroquinolones	Ciprofloxacin 400 8 hourly
Colistin	9-12 MU (720-960 mg) loading dose followed by 480 mg 12 hourly if patient without kidney injury.
Beta-lactams	Cefepime 2 g 8 hourly
	Ceftazidime 2 g 6 hourly
	Imipenem 1 g 6-8 hourly
	Meropenem 1 g 6-8 hourly
	Ertapenem 1 g 12 hourly
	Piperacillin-Tazobactam 4.5 g 6 hourly
Glycopeptides	Vancomycin 35 mg/kg in a 1 hour infusion loading dose followed by 30 mg/kg continuous infusion
Daptomycin	8-12 mg/kg 24 hourly
Tigecyclin	200 mg followed by 100 mg 12 hourly when borderline susceptibility is suspected

For time-dependent antimicrobials such as beta lactams or glycopeptides, the use of continuous or extended infusions of antimicrobial is able to increase the fT > MIC. Continuous infusion required the antimicrobial to be stable after preparation at room air temperature. It has been tested successfully with ceftazidime, cefepime, piperacillin-tazobactam, doripenem, meropenem and vancomycin. The clinical proof of superiority of extended or continuous infusion of betalactams or vancomycin is suggested by few studies [64, 67–71], and required further trials.

For concentration dependent antimicrobials, such as aminoglycosides [72, 73] or colistin [65, 66], the use of increased doses increased the probability to reach the pharmacodynamic targets.

Therapeutic drug monitoring (TDM) is common place, given uncertainties on the antimicrobial concentration actually achieved, especially for aminoglycosides and glycopeptides. Recent data have also shown that TDM of beta-lactams leads to dose adjustment in about half of cases [63, 74].

In case of kidney injury, hydrophylic antibiotics clearance will be largely modified. Intensity of continuous renal replacement therapy and degree of residual renal function are crucial factors in accurately determining the antimicrobial requirement.

### Right after the initiation of the antimicrobial therapy

#### Results of microbiological exams

Despite all previous knowledge, antibiotic therapy remained initially inadequate in 20-40% of the cases, even if broad spectrum combination therapy is used. Microbiological samples, collected just before antimicrobial start, may help to document the infection and decrease the risk of inadequate treatment. Good collaboration with the microbiological laboratory, Gram stain examination and new molecular techniques able to detect early various antimicrobial resistance mechanisms are important strategies to be developed in order to shorten the delay for appropriate treatment and subsequent de-escalation [75–78].

### Source control

Source control is recognized as an important part of the treatment of BSIs and has been recently shown to be independently related with outcome [12, 79]. The source control should be optimal, especially when microorganisms are less susceptible to antimicrobials or when antimicrobial diffusion is hazardous.

### During the antimicrobial therapy

#### De-escalation

Antimicrobial de-escalation is a clinical approach to empirical antibiotic treatment of bacteremia that attempts to balance the need for appropriate initial therapy with the need to limit unnecessary antimicrobial exposure, in order to curtail the emergence of resistance.

Paradoxically, although de-escalation is associated with no or a positive impact on mortality [80], its impact on overall antibiotic consumption has not been clearly demonstrated. It has even been associated with an increase in the number of days on antibiotics [81]. However, it clearly reduced the broad-spectrum antibiotic use. The value of such streamlining may seem intuitive to infectious diseases physicians, but for many other physicians, an attitude of “don’t change a winning team” is pervasive. The persistently reported figures of around 50% de-escalation delivery are unacceptable and must be improved. The requirement for multiple antimicrobials to achieve adequate initial therapy further emphasizes the need for obtaining both timely blood culture and early culture results through incorporation of molecular diagnostics for bacteremia [78, 82] so that subsequent antimicrobial de-escalation can be performed in a rational manner [82].

### Length of antimicrobial therapy

Up to half of the antibiotic use is unnecessary or inappropriate, and excessive duration is the greatest contributor to inappropriate use. A reduction in the length of antibiotic courses in ICU is an important strategy to minimize the consequences of antimicrobial overuse, including antibiotic resistance, *Clostridium Difficile*-associated diarrhea, adverse effects and costs. Usual length of therapy is about 10 days in routine, longer for *S. aureus* bacteremia and shorter for coagulase-negative staphylococci bacteremia [83].

Although large RCTs are missing, a meta-analysis of available data suggested that a short (5-7 days) therapy, vs. a longer one (7-21 days), is safe with respect to clinical or microbiologic cure and survival [6]. Of note, aminoglycosides treatment should be limited to 2-5 days to optimize efficacy and to limit potential toxicity.

Of course, at the bedside, many factors should be taken into account to decide to shorten the duration of treatment (Table 4). A short duration of treatment should be more cautiously decided when the initial treatment was inadequate [84], when the therapeutic index was low, when the source control was not optimal. In immunocompromised patients, and when invasive devices or foreign material are left in place, a longer treatment is often recommended.

**Table 4:** Arguments pro and against a short duration of antibiotic therapy

Arguments for a short treatment	Arguments for a longer treatment
No comorbid conditions	Immune depression
Source control	No source control
Low MICs, high bactericidal titers	MDR , XDR bacterias
Initial appropriate therapy	Low bactericidal titers
Easy PK and tissue diffusion	Poor PK and tissue diffusion
Source control appropriate	Foreign materials
No foreign material	Slow, partial clinical response
Rapid clinical improvement	

To reduce the duration of treatment, strategy should also be guided by the evolution of patients’ status under treatment and by biomarkers [85]. Procalcitonin (PCT) serum level has been used in randomized control trial to reduce the duration of therapy in severe infections. A PCT-driven approach was responsible for an increase of 3.5 of the number of days alive without antibiotics at day 28, with no adverse impact, as compared to the standard of care. Surprisingly, the PCT rules are not followed by clinicians in about half of the cases, although it suggests that regular monitoring of PCT may serve as a target to propose stopping antimicrobials.

The use of external rules of therapy, of systematic intervention of the antibiotic team of the hospital [86, 87] or bundles [88], simple educational programs with audit, are also effective in reducing the antimicrobial consumption to a similar extent without adverse events.

Finally individual patient therapy needs to be coupled with adherence to optimal prevention practice to prevent spread of resistant bacteria from patient to patient.

## Conclusions

Bacteremia is an independent risk factor of mortality in case of severe sepsis in ICU. Early adequate treatment of bloodstream infections is required and is based on previous knowledge and guidelines, rapid microbiological identification, adequate source control and administration of proper antimicrobials at the adequate dose regimen for the individual patient. These goals become difficult to achieve in case of BSI due to multi-drug resistant pathogens with high MICs to antimicrobials, in the most severe patients where low antibiotic concentrations are common, and when the source control is incomplete or impossible. In these situations, pharmacodynamic optimization and therapeutic drug monitoring should be recommended.

## Authors’ original submitted files for images

Below are the links to the authors’ original submitted files for images. Authors’ original file for figure 1

## References

[1] Timsit JF, Laupland KB (2012). Update on bloodstream infections in ICUs. Curr Opin Crit Care.

[2] Laupland KB, Gregson DB, Zygun DA, Doig CJ, Mortis G, Church DL (2004). Severe bloodstream infections: a population-based assessment. Crit Care Med.

[3] Vincent JL, Rello J, Marshall J, Silva E, Anzueto A, Martin CD (2009). International study of the prevalence and outcomes of infection in intensive care units. JAMA.

[4] Prowle JR, Echeverri JE, Ligabo EV, Sherry N, Taori GC, Crozier TM (2011). Acquired bloodstream infection in the intensive care unit: incidence and attributable mortality. Crit Care (London, England).

[5] Carlet J, Collignon P, Goldmann D, Goossens H, Gyssens IC, Harbarth S (2011). Society’s failure to protect a precious resource: antibiotics. Lancet.

[6] Havey TC, Fowler RA, Daneman N (2011). Duration of antibiotic therapy for bacteremia: a systematic review and meta-analysis. Crit Care (London, England).

[7] Bouadma L, Luyt CE, Tubach F, Cracco C, Alvarez A, Schwebel C (2010). Use of procalcitonin to reduce patients’ exposure to antibiotics in intensive care units (PRORATA trial): a multicentre randomised controlled trial. Lancet.

[8] Schuetz P, Chiappa V, Briel M, Greenwald JL (2011). Procalcitonin algorithms for antibiotic therapy decisions: a systematic review of randomized controlled trials and recommendations for clinical algorithms. Arch Intern Med.

[9] Retamar P, Portillo MM, Lopez-Prieto MD, Rodriguez-Lopez F, de Cueto M, Garcia MV (2012). Impact of inadequate empirical therapy on the mortality of patients with bloodstream infections: a propensity score-based analysis. Antimicrob Agents Chemother.

[10] Kumar A, Ellis P, Arabi Y, Roberts D, Light B, Parrillo JE (2009). Initiation of inappropriate antimicrobial therapy results in a fivefold reduction of survival in human septic shock. Chest.

[11] Ferrer R, Martin-Loeches I, Phillips G, Osborn TM, Townsend S, Dellinger RP (2014). Empiric antibiotic treatment reduces mortality in severe sepsis and septic shock from the first hour: results from a guideline-based performance improvement program. Crit Care Med.

[12] Tabah A, Koulenti D, Laupland K, Misset B, Valles J, Bruzzi de Carvalho F (2012). Characteristics and determinants of outcome of hospital-acquired bloodstream infections in intensive care units: the EUROBACT International Cohort Study. Intensive Care Med.

[13] Puskarich MA, Trzeciak S, Shapiro NI, Arnold RC, Horton JM, Studnek JR (2011). Association between timing of antibiotic administration and mortality from septic shock in patients treated with a quantitative resuscitation protocol. Crit Care Med.

[14] Yealy DM, Kellum JA, Huang DT, Barnato AE, Weissfeld LA, Pike F (2014). A randomized trial of protocol-based care for early septic shock. N Engl J Med.

[15] Woerther PL, Burdet C, Chachaty E, Andremont A (2013). Trends in human fecal carriage of extended-spectrum beta-lactamases in the community: toward the globalization of CTX-M. Clin Microbiol Rev.

[16] Tham J, Odenholt I, Walder M, Brolund A, Ahl J, Melander E (2010). Extended-spectrum beta-lactamase-producing Escherichia coli in patients with travellers’ diarrhoea. Scand J Infect Dis.

[17] Peirano G, Laupland KB, Gregson DB, Pitout JD (2011). Colonization of returning travelers with CTX-M-producing Escherichia coli. J Travel Med.

[18] Stralin K, Soderquist B (2006). Staphylococcus aureus in community-acquired pneumonia. Chest.

[19] Woodhead M, Blasi F, Ewig S, Garau J, Huchon G, Ieven M (2011). Guidelines for the management of adult lower respiratory tract infections–full version. Clin Microbiol Infect.

[20] Nathwani D, Morgan M, Masterton RG, Dryden M, Cookson BD, French G (2008). Guidelines for UK practice for the diagnosis and management of methicillin-resistant Staphylococcus aureus (MRSA) infections presenting in the community. J Antimicrob Chemother.

[21] Leone M, Bourgoin A, Cambon S, Dubuc M, Albanese J, Martin C (2003). Empirical antimicrobial therapy of septic shock patients: adequacy and impact on the outcome. Crit Care Med.

[22] Zahar JR, Timsit JF, Garrouste-Orgeas M, Francais A, Vesin A, Descorps-Declere A (2011). Outcomes in severe sepsis and patients with septic shock: pathogen species and infection sites are not associated with mortality. Crit Care Med.

[23] Marra AR, Camargo LF, Pignatari AC, Sukiennik T, Behar PR, Medeiros EA (2011). Nosocomial bloodstream infections in Brazilian hospitals: analysis of 2,563 cases from a prospective nationwide surveillance study. J Clin Microbiol.

[24] Valles J, Alvarez-Lerma F, Palomar M, Blanco A, Escoresca A, Armestar F (2011). Health-care-associated bloodstream infections at admission to the ICU. Chest.

[25] Corona A, Bertolini G, Lipman J, Wilson AP, Singer M (2010). Antibiotic use and impact on outcome from bacteraemic critical illness: the BActeraemia Study in Intensive Care (BASIC). J Antimicrob Chemother.

[26] Souli M, Galani I, Giamarellou H (2008). Emergence of extensively drug-resistant and pandrug-resistant Gram-negative bacilli in Europe. Euro Surveill.

[27] Chung DR, Song JH, Kim SH, Thamlikitkul V, Huang SG, Wang H (2011). High prevalence of multidrug-resistant nonfermenters in hospital-acquired pneumonia in Asia. Am J Respir Crit Care Med.

[28] Lye DC, Earnest A, Ling ML, Lee TE, Yong HC, Fisher DA (2012). The impact of multidrug resistance in healthcare-associated and nosocomial Gram-negative bacteraemia on mortality and length of stay: cohort study. Clin Microbiol Infect.

[29] Garner JS, Jarvis WR, Emori TG, Horan TC, Hughes JM (1988). CDC definitions for nosocomial infections, 1988. Am J Infect Control.

[30] Friedman ND, Kaye KS, Stout JE, McGarry SA, Trivette SL, Briggs JP (2002). Health care–associated bloodstream infections in adults: a reason to change the accepted definition of community-acquired infections. Ann Intern Med.

[31] Valles J, Calbo E, Anoro E, Fontanals D, Xercavins M, Espejo E (2008). Bloodstream infections in adults: importance of healthcare-associated infections. J Infect.

[32] De Bus L, Coessens G, Boelens J, Claeys G, Decruyenaere J, Depuydt P (2013). Microbial etiology and antimicrobial resistance in healthcare-associated versus community-acquired and hospital-acquired bloodstream infection in a tertiary care hospital. Diagn Microbiol Infect Dis.

[33] Klompas M, Yokoe DS (2009). Automated surveillance of health care-associated infections. Clin Infect Dis.

[34] Kollef MH, Zilberberg MD, Shorr AF, Vo L, Schein J, Micek ST (2011). Epidemiology, microbiology and outcomes of healthcare-associated and community-acquired bacteremia: a multicenter cohort study. J Infect.

[35] Lenz R, Leal JR, Church DL, Gregson DB, Ross T, Laupland KB (2012). The distinct category of healthcare associated bloodstream infections. BMC Infect Dis.

[36] Marschall J, Fraser VJ, Doherty J, Warren DK (2009). Between community and hospital: healthcare-associated gram-negative bacteremia among hospitalized patients. Infect Control Hosp Epidemiol.

[37] Rodriguez-Bano J, Lopez-Prieto MD, Portillo MM, Retamar P, Natera C, Nuno E (2010). Epidemiology and clinical features of community-acquired, healthcare-associated and nosocomial bloodstream infections in tertiary-care and community hospitals. Clin Microbiol Infect.

[38] Johnson MT, Reichley R, Hoppe-Bauer J, Dunne WM, Micek S, Kollef M (2011). Impact of previous antibiotic therapy on outcome of Gram-negative severe sepsis. Crit Care Med.

[39] Harbarth S, Garbino J, Pugin J, Romand JA, Lew D, Pittet D (2003). Inappropriate initial antimicrobial therapy and its effect on survival in a clinical trial of immunomodulating therapy for severe sepsis. Am J Med.

[40] Planquette B, Timsit JF, Misset BY, Schwebel C, Azoulay E, Adrie C (2013). Pseudomonas aeruginosa ventilator-associated pneumonia. predictive factors of treatment failure. Am J Respir Crit Care Med.

[41] Routsi C, Pratikaki M, Platsouka E, Sotiropoulou C, Papas V, Pitsiolis T (2013). Risk factors for carbapenem-resistant Gram-negative bacteremia in intensive care unit patients. Intensive Care Med.

[42] Armand-Lefevre L, Angebault C, Barbier F, Hamelet E, Defrance G, Ruppe E (2013). Emergence of imipenem-resistant gram-negative bacilli in intestinal flora of intensive care patients. Antimicrob Agents Chemother.

[43] Adrie C, Azoulay E, Francais A, Clec’h C, Darques L, Schwebel C (2007). Influence of gender on the outcome of severe sepsis: a reappraisal. Chest.

[44] Adrie C, Schwebel C, Garrouste-Orgeas M, Vignoud L, Planquette B, Azoulay E (2013). Initial use of one or two antibiotics for critically ill patients with community-acquired pneumonia: impact on survival and bacterial resistance. Crit Care (London, England).

[45] von Baum H, Welte T, Marre R, Suttorp N, Ewig S (2010). Community-acquired pneumonia through Enterobacteriaceae and Pseudomonas aeruginosa: Diagnosis, incidence and predictors. Eur Respir J.

[46] Timsit JF, L’Heriteau F, Lepape A, Francais A, Ruckly S, Venier AG (2012). A multicentre analysis of catheter-related infection based on a hierarchical model. Intensive Care Med.

[47] Timsit JF, Tabah A, Koulenti D, Ruckly S, Laupland KB, Garrouste Orgeas M (2014). Update in hospital-acquired bacteremia respiratory infections: experience from the EUROBACT study. Clin Pulmon Med.

[48] De Waele JJ, Hoste EA, Blot SI (2008). Blood stream infections of abdominal origin in the intensive care unit: characteristics and determinants of death. Surg Infect (Larchmt).

[49] Solomkin JS, Mazuski JE, Bradley JS, Rodvold KA, Goldstein EJ, Baron EJ (2010). Diagnosis and management of complicated intra-abdominal infection in adults and children: guidelines by the Surgical Infection Society and the Infectious Diseases Society of America. Clin Infect Dis.

[50] Spellberg B, Blaser M, Guidos RJ, Boucher HW, Bradley JS, Eisenstein BI (2011). Combating antimicrobial resistance: policy recommendations to save lives. Clin Infect Dis.

[51] Stevens DL, Bisno AL, Chambers HF, Dellinger EP, Goldstein EJ, Gorbach SL (2014). Practice guidelines for the diagnosis and management of skin and soft tissue infections: 2014 update by the Infectious Diseases Society of America. Clin Infect Dis.

[52] Guidelines for the management of adults with hospital-acquired, ventilator-associated, and healthcare-associated pneumonia. Am J Respir Crit Care Med. 2005, 171 (4): 388-416. Epub 2005/02/09 10.1164/rccm.200405-644ST 15699079

[53] Hooton TM, Bradley SF, Cardenas DD, Colgan R, Geerlings SE, Rice JC (2010). Diagnosis, prevention, and treatment of catheter-associated urinary tract infection in adults: 2009 International Clinical Practice Guidelines from the Infectious Diseases Society of America. Clin Infect Dis.

[54] Dellinger RP, Levy MM, Rhodes A, Annane D, Gerlach H, Opal SM (2013). Surviving Sepsis Campaign: international guidelines for management of severe sepsis and septic shock, 2012. Intensive Care Med.

[55] Mermel LA, Allon M, Bouza E, Craven DE, Flynn P, O’Grady NP (2009). Clinical practice guidelines for the diagnosis and management of intravascular catheter-related infection: 2009 Update by the Infectious Diseases Society of America. Clin Infect Dis.

[56] Brunkhorst FM, Oppert M, Marx G, Bloos F, Ludewig K, Putensen C (2012). Effect of empirical treatment with moxifloxacin and meropenem vs meropenem on sepsis-related organ dysfunction in patients with severe sepsis: a randomized trial. JAMA.

[57] Kumar A, Safdar N, Kethireddy S, Chateau D (2010). A survival benefit of combination antibiotic therapy for serious infections associated with sepsis and septic shock is contingent only on the risk of death: a meta-analytic/meta-regression study. Crit Care Med.

[58] Pena C, Suarez C, Ocampo-Sosa A, Murillas J, Almirante B, Pomar V (2013). Effect of adequate single-drug vs combination antimicrobial therapy on mortality in Pseudomonas aeruginosa bloodstream infections: a post Hoc analysis of a prospective cohort. Clin Infect Dis.

[59] Micek ST, Welch EC, Khan J, Pervez M, Doherty JA, Reichley RM (2010). Empiric combination antibiotic therapy is associated with improved outcome against sepsis due to Gram-negative bacteria: a retrospective analysis. Antimicrob Agents Chemother.

[60] Martinez JA, Cobos-Trigueros N, Soriano A, Almela M, Ortega M, Marco F (2010). Influence of empiric therapy with a beta-lactam alone or combined with an aminoglycoside on prognosis of bacteremia due to gram-negative microorganisms. Antimicrob Agents Chemother.

[61] Udy AA, Roberts JA, Lipman J (2013). Clinical implications of antibiotic pharmacokinetic principles in the critically ill. Intensive Care Med.

[62] Esterly JS, Wagner J, McLaughlin MM, Postelnick MJ, Qi C, Scheetz MH (2012). Evaluation of clinical outcomes in patients with bloodstream infections due to Gram-negative bacteria according to carbapenem MIC stratification. Antimicrob Agents Chemother.

[63] Roberts JA, Paul SK, Akova M, Bassetti M, De Waele JJ, Dimopoulos G (2014). DALI: defining antibiotic levels in intensive care unit patients: Are current beta-lactam antibiotic doses sufficient for critically Ill patients?. Clin Infect Dis.

[64] Beumier M, Roberts JA, Kabtouri H, Hites M, Cotton F, Wolff F (2013). A new regimen for continuous infusion of vancomycin during continuous renal replacement therapy. J Antimicrob Chemother.

[65] Garonzik SM, Li J, Thamlikitkul V, Paterson DL, Shoham S, Jacob J (2011). Population pharmacokinetics of colistin methanesulfonate and formed colistin in critically ill patients from a multicenter study provide dosing suggestions for various categories of patients. Antimicrob Agents Chemother.

[66] Mohamed AF, Karaiskos I, Plachouras D, Karvanen M, Pontikis K, Jansson B (2012). Application of a loading dose of colistin methanesulfonate in critically ill patients: population pharmacokinetics, protein binding, and prediction of bacterial kill. Antimicrob Agents Chemother.

[67] Falagas ME, Tansarli GS, Ikawa K, Vardakas KZ (2013). Clinical outcomes with extended or continuous versus short-term intravenous infusion of carbapenems and piperacillin/tazobactam: a systematic review and meta-analysis. Clin Infect Dis.

[68] Dulhunty JM, Roberts JA, Davis JS, Webb SA, Bellomo R, Gomersall C (2013). Continuous infusion of beta-lactam antibiotics in severe sepsis: a multicenter double-blind, randomized controlled trial. Clin Infect Dis.

[69] Dulhunty JM, Roberts JA, Davis JS, Webb SA, Bellomo R, Gomersall C (2013). A protocol for a multicentre randomised controlled trial of continuous beta-lactam infusion compared with intermittent beta-lactam dosing in critically ill patients with severe sepsis: the BLING II study. Crit Care Resusc.

[70] Lodise TP, Lomaestro B, Drusano GL (2007). Piperacillin-tazobactam for Pseudomonas aeruginosa infection: clinical implications of an extended-infusion dosing strategy. Clin Infect Dis.

[71] Wysocki M, Delatour F, Faurisson F, Rauss A, Pean Y, Misset B (2001). Continuous versus intermittent infusion of vancomycin in severe Staphylococcal infections: prospective multicenter randomized study. Antimicrob Agents Chemother.

[72] de Montmollin E, Bouadma L, Gault N, Mourvillier B, Mariotte E, Chemam S (2014). Predictors of insufficient amikacin peak concentration in critically ill patients receiving a 25 mg/kg total body weight regimen. Intensive Care Med.

[73] Duszynska W, Taccone FS, Hurkacz M, Kowalska-Krochmal B, Wiela-Hojenska A, Kubler A (2013). Therapeutic drug monitoring of amikacin in septic patients. Crit Care (London, England).

[74] Seyler L, Cotton F, Taccone FS, De Backer D, Macours P, Vincent JL (2011). Recommended beta-lactam regimens are inadequate in septic patients treated with continuous renal replacement therapy. Crit Care (London, England).

[75] Huang AM, Newton D, Kunapuli A, Gandhi TN, Washer LL, Isip J (2013). Impact of rapid organism identification via matrix-assisted laser desorption/ionization time-of-flight combined with antimicrobial stewardship team intervention in adult patients with bacteremia and candidemia. Clin Infect Dis.

[76] Romero-Gomez MP, Gomez-Gil R, Pano-Pardo JR, Mingorance J (2012). Identification and susceptibility testing of microorganism by direct inoculation from positive blood culture bottles by combining MALDI-TOF and Vitek-2 Compact is rapid and effective. J Infect.

[77] Vlek AL, Bonten MJ, Boel CH (2012). Direct matrix-assisted laser desorption ionization time-of-flight mass spectrometry improves appropriateness of antibiotic treatment of bacteremia. PLoS One.

[78] Clerc O, Prod’hom G, Vogne C, Bizzini A, Calandra T, Greub G (2013). Impact of matrix-assisted laser desorption ionization time-of-flight mass spectrometry on the clinical management of patients with Gram-negative bacteremia: a prospective observational study. Clin Infect Dis.

[79] Boyer A, Vargas F, Coste F, Saubusse E, Castaing Y, Gbikpi-Benissan G (2009). Influence of surgical treatment timing on mortality from necrotizing soft tissue infections requiring intensive care management. Intensive Care Med.

[80] Garnacho-Montero J, Gutierrez-Pizarraya A, Escoresca-Ortega A, Corcia-Palomo Y, Fernandez-Delgado E, Herrera-Melero I (2014). De-escalation of empirical therapy is associated with lower mortality in patients with severe sepsis and septic shock. Intensive Care Med.

[81] Shime N, Satake S, Fujita N (2011). De-escalation of antimicrobials in the treatment of bacteraemia due to antibiotic-sensitive pathogens in immunocompetent patients. Infection.

[82] Kollef MH (2014). What can be expected from antimicrobial de-escalation in the critically ill?. Intensive Care Med.

[83] Daneman N, Shore K, Pinto R, Fowler R (2011). Antibiotic treatment duration for bloodstream infections in critically ill patients: a national survey of Canadian infectious diseases and critical care specialists. Int J Antimicrob Agents.

[84] Kollef MH, Chastre J, Clavel M, Restrepo MI, Michiels B, Kaniga K (2012). A randomized trial of 7-day doripenem versus 10-day imipenem-cilastatin for ventilator-associated pneumonia. Crit Care (London, England).

[85] Matthaiou DK, Ntani G, Kontogiorgi M, Poulakou G, Armaganidis A, Dimopoulos G (2012). An ESICM systematic review and meta-analysis of procalcitonin-guided antibiotic therapy algorithms in adult critically ill patients. Intensive Care Med.

[86] Zahar JR, Rioux C, Girou E, Hulin A, Sauve C, Bernier-Combes A (2006). Inappropriate prescribing of aminoglycosides: risk factors and impact of an antibiotic control team. J Antimicrob Chemother.

[87] Fries BL, Licitra C, Crespo A, Akhter K, Busowski MT, Salazar D (2014). Infectious diseases consultation and the management of Staphylococcus aureus bacteremia. Clin Infect Dis.

[88] Lopez-Cortes LE, Del Toro MD, Galvez-Acebal J, Bereciartua-Bastarrica E, Farinas MC, Sanz-Franco M (2013). Impact of an evidence-based bundle intervention in the quality-of-care management and outcome of Staphylococcus aureus bacteremia. Clin Infect Dis.

[CR89] The pre-publication history for this paper can be accessed here:http://www.biomedcentral.com/1471-2334/14/489/prepub

