# Supplementary material for: Treatment of bloodstream infections in ICUs
Source: BMC Infect Dis. 2014 Nov 28;14:489. doi: 10.1186/1471-2334-14-489 (PMC4289315; doi:10.1186/1471-2334-14-489)
Supplement: Supplementary file 1 — Authors’ original file for figure 1 [file 12879_2014_4065_MOESM1_ESM.pptx]

## Slide 1
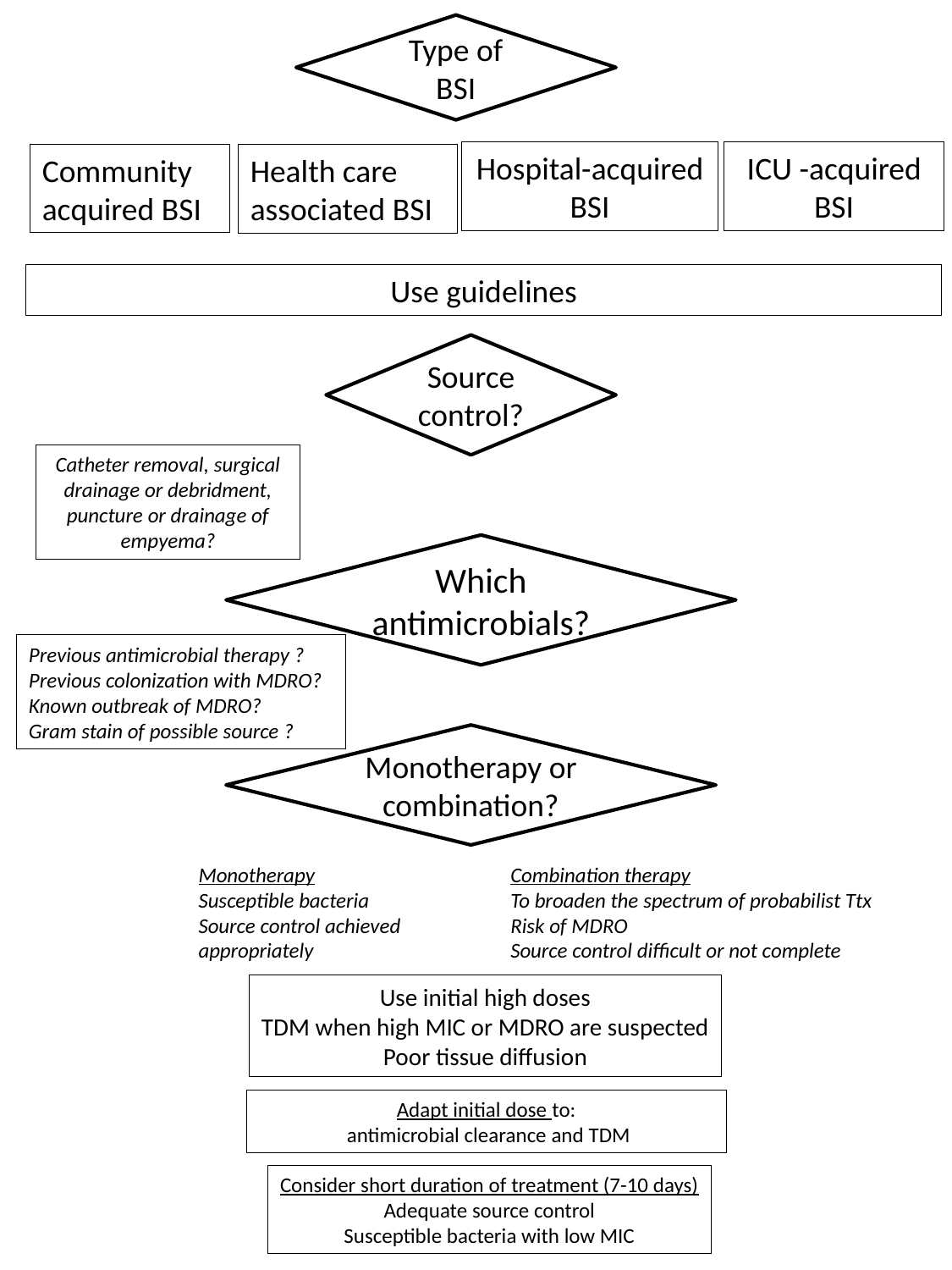

Type of BSI
Hospital-acquired BSI
ICU -acquired BSI
Community acquired BSI
Health care associated BSI
Use guidelines
Source control?
Catheter removal, surgical drainage or debridment, puncture or drainage of empyema?
Which antimicrobials?
Previous antimicrobial therapy ?
Previous colonization with MDRO?
Known outbreak of MDRO?
Gram stain of possible source ?
Monotherapy or combination?
Monotherapy
Susceptible bacteria
Source control achieved appropriately
Combination therapy
To broaden the spectrum of probabilist Ttx
Risk of MDRO
Source control difficult or not complete
Use initial high doses
TDM when high MIC or MDRO are suspected
Poor tissue diffusion
Adapt initial dose to:
 antimicrobial clearance and TDM
Consider short duration of treatment (7-10 days)
Adequate source control
Susceptible bacteria with low MIC
